# Juvenile dermatomyositis in South African children is characterised by frequent dystropic calcification: a cross sectional study

**DOI:** 10.1186/1546-0096-12-2

**Published:** 2014-01-07

**Authors:** Gail Faller, Bhadrish J Mistry, Mohammed Tikly

**Affiliations:** 1 Division of Rheumatology, Department of Paediatrics, Chris Hani Baragwanath Hospital and the University of the Witwatersrand, Johannesburg, South Africa; 2 Division of Rheumatology Internal Medicine, Faculty of Health Sciences, Chris Hani Baragwanath Hospital and the University of the Witwatersrand, Johannesburg, South Africa

**Keywords:** Juvenile dermatomyositis, Africa, Black, Calcinosis, Staphylococcus aureus

## Abstract

**Background:**

To describe Juvenile dermatomyositis (JDM) that has rarely been reported in Sub-Saharan Africa in children.

**Methods:**

Retrospective record review of children with JDM attending a tertiary hospital in South Africa.

**Results:**

Twenty-one children (16 female, five male) with JDM had a mean (SD) age at presentation of 9.8 (3.3) years. Mean follow-up period was 2.6 (2.2) years. The commonest presenting features were skin rash (71%), muscle weakness (71%), inflammatory arthritis (42%) and calcinosis (29%). The cumulative frequency of calcinosis was 71%. Skin vasculitis was present in 9(43%), and 7 (33%) had *Staphylococcus aureus* infections. Calcinosis was strongly associated with vasculitis; 11/15 (73.3%) with calcinosis had vasculitis versus 0/6 without vasculitis (p = 0.003). Patients with calcinosis had significantly lower creatinine kinase (CK) levels compared to those without calcinosis [mean (SD) 272 U/L (401) vs. 2414 U/L (3201), respectively, p = 0.016]. All children with calcinosis had *Staphylococcus aureus* infection, but there was no significant difference in their duration of symptoms to presentation. Joint contractures, occurring in eight patients (38%), were associated with a significantly lower age at presentation [mean (SD) 6.8(2.8) vs. 11.6(2.1) years (no contractures) p = 0.0003], and significantly higher CRP and ESR levels. Three patients were lost to follow-up, two died. In the remaining 16 patients: 10 (47%) experienced remission, 2 relapsed and 4 persistent active disease.

**Conclusion:**

African children with JDM have increased vasculitic disease and high levels of calcinosis with low muscle enzymes, particularly CK. Younger children are at higher risk of contractures and disability. Patients are at high risk of developing *Staphylococcus aureus* infection. Rapid and aggressive therapy is necessary.

## Background

Juvenile dermatomyositis (JDM) is a systemic capillary vasculopathy affecting primarily the skin and muscles and less frequently the gastrointestinal tract, myocardium and lungs. Clinical presentation varies from a catastrophic presentation of severe weakness (or paralysis), swallowing abnormalities and myocarditis, to a more insidious onset of muscle weakness [1]. Diagnosis is still largely based on diagnostic criteria of Bohan and Peter [2,3], but more recently MRI features suggestive an inflammatory myositis have been used [4] and an international consensus study network for juvenile dermatomyositis has proposed other useful criteria including calcinosis, dysphonia, nailfold capillary changes and myopathic changes on EMG [5].

Juvenile dermatomyositis is rare with an incidence of 1.9-4.1 per million in industrialised countries. It has a female preponderance across all age groups [6,7]. While regional differences are known to play a role, it is unknown whether it is more common in people of African extraction [8]. In recent years, mortality due to JDM has declined considerably but morbidity related to the myopathy, calcinosis and long-term use of corticosteroids remains a major challenge [9].

To date there have been no published reports of JDM in Sub-Saharan Africans. We therefore undertook a retrospective study of the clinical presentation and outcome of JDM in South African children attending a tertiary hospital. The study was approved by Human Ethics Committee of the Faculty of Health Sciences, University of the Witwatersrand, Johannesburg, South Africa.

## Methods

A retrospective record review was conducted of children and adolescents who fulfilled the Bohan and Peter criteria [2,3] for JDM and were diagnosed between January 1998 and December 2009. The patients were attending the paediatric and adult rheumatology clinics at the Chris Hani Academic Baragwanath Academic Hospital, Johannesburg, South Africa. This is a tertiary care center serving the predominantly Black population of Soweto and surrounding areas. The paediatric rheumatology clinic is the larger of only 2 paediatric rheumatology clinics in the northern part of South Africa, and most patients with rheumatological conditions from district hospitals, primary care facilities and paediatricians are referred to this clinic. Data abstracted from case notes included demographics, clinical and laboratory features at diagnosis, medical treatment and outcome. As data concerning muscle biopsies was not always available, this was not included, and routine capillaroscopy was not performed. The subtypes of calcinosis were defined according to the classification by Blane et al. [10]. Global weakness was defined as weakness that extended beyond the usual muscles identified as most often affected in JDM (that is, the hip flexors, hip extensors, hip abductors, neck flexors and shoulder abductors) and therefore included peripheral weakness (hands and feet) and abdominal muscles [11]. Weakness was assessed by standard manual muscle testing.

### Statistical methods

The Students t-test was applied to compare continuous variables and the two-tailed Fisher’s exact test in the case of categorical variables. All statistical analyses were done using the Statistica v10 software [12]. A p value <0.05 was considered to be significant.

## Results

All the patients were Black Africans and, as shown in Table 1, more than 75% of the 21 patients were female. The mean age at presentation was just under 10 years with a mean follow-up period of 31.6 months. At presentation, the commonest features were skin rashes (Gottron’s papules, heliotrope or malar rash or a combination thereof) in 90%, and proximal muscle weakness and arthritis each in 48% of patients. Although 10% of children were not described to have typical rashes at presentation, these were identified during the follow-up course.

**Table 1:** Demographic and clinical features of South African children with dermatomyositis

**Clinical features**	**At presentation (%)**	**Cumulative frequency (%)**
F:M ratio	16:5	
Mean (SD) age in years	9.8.(3.3)	
Mean (SD) follow-up in months		31.6(27.46)
Rash	19/21(90)	19/21(90)
Proximal weakness	10/21(48)	10/21(48)
Global weakness	5/21(24)	5/21(24)
Arthritis	10/21(48)	11/21(52)
Contractures	4/21(19)	8/21(38)
Calcinosis	7/21(33)	15/21(71)
Vasculitis	9/21(43)	11/21(52)
*Staphylococcus aureus* infection	1/21(5)	7/21(33)
Interstitial lung disease	1/21(5)	2/21(10)

As shown in Table 2, patients with global weakness were younger (p = 0.04), had higher baseline acute phase responses, CRP (p = 0.03) and ESR (p = 0.007). Joint Contractures (defined as limited range of movement,) occurred in 8 patients, were associated with a younger age of disease onset (p = 0.003) and higher baseline acute phase responses: CRP (p = 0.01) and ESR (p = 0.04). Two of the 5 patients with global weakness had associated dysphagia necessitating naso-gastric tube feeding, 1 of whom was also in congestive cardiac failure.

**Table 2:** Clinico-laboratory correlates

***Global weakness***	***Present (n = 5)***	***Absent (n = 16)***	***p value***
Age - mean (SD) years	7.3(2)	10.5(3.3)	0.04
CRP - mean (SD) mg/l	52(81)	4(4.5)	0.03
ESR - mean (SD) mm/hr	83(41)	24(25)	0.007
***Contractures***	***Present (n = 8)***	***Absent (n = 13)***	
Age - mean (SD) years	6.8(3)	11.6(2)	0.0003
CRP - mean (SD) mg/l	45(75)	3.8(3.9)	0.000
ESR - mean (SD) mm/hr	59(48)	25(26)	0.04
***Calcinosis***	***Present (n = 15)***	***Absent (n = 6)***	
Age-mean (SD) years	9.1(3.5)	11.3(2)	0.18
Duration of follow-up - mean (SD) (months)	39 (28)	13(14)	0.04
CK - mean (SD) IU/l	288 (402)	2414(3201)	0.02
Aldolase mean (SD) IU/	18.5 (12.6)	38.6(23.5)	0.03
Vasculitis (%)	11(73.3)	0 (0)	0.003
***Staphylococcus aureus*** infection (%)	7 (46,7)	0 (0)	0.04
***Staphylococcus aureus infection***	***Present (n = 7)***	***Absent (n = 14)***	
Duration of follow-up - mean (SD) (months)	51(32)	22(20)	0.02

A third of the patients had calcinosis at presentation and a further 38% developed this complication during the follow-up period. The subtypes of calcinosis were superficial nodules in 4 (26.6%), deep muscle tumoural calcification in 5, (33.3%), and mixed calcification types in the remaining 6 (40%) including along fascial planes and exoskeleton formation. The mean duration of symptoms of disease prior to presentation in patients with calcinosis (as described by their care-givers) was 9.2, (9.4) months compared to those who did not develop calcinosis at 4.5 [3] months. This difference was not statistically significant (p = 0.24).

The presence of skin vasculitis, as defined by the presence of vasculopathic lesions with cutaneous ulceration, had the strongest association with calcinosis (p = 0.003) (Table 2). The muscle enzymes, CK and aldolase, were significantly lower in patients with calcinosis (p = 0.02 and 0.003, respectively).

Vasculitis and *Staphylococcus aureus* infection occurred exclusively in patients with calcinosis.

*Staphylococcus aureus* infection occurred in one third of patients, all of whom had calcinosis; in 4 the infection presented as abscess formation in association with the calcification, in 2 with septic arthritis (1 with osteomyelitis) and 1 had septicaemia. In all these patient positive cultures were obtained from pus swabs or blood cultures. The patients who developed *Staphylococcus aureus* infection had a significantly longer follow-up period (p = 0.01).

Interstitial lung disease occurred in 2 (12%), both of whom developed cor pulmonale. Two patients contracted tuberculosis (TB), 1 pulmonary and the other TB arthritis. The diagnosis in both cases was confirmed by Mycobacterium Tuberculosis culture. One child had uveitis at presentation and another developed it during the disease course.

### Laboratory features

Only five patients were antinuclear antibody (ANA) positive and all except one had elevation of at least one muscle enzyme, with aldolase abnormal in 18/21 (85%) and creatinine kinase (CK) in 10/21 (48%). Lactate dehydrogenase (LDH) was not done routinely but was raised in 7 of 9 children tested (33%). There was lymphopenia (total count less than 1.5 × 10^9^) in six patients (29%). Baseline erythrocyte sedimentation rate (ESR) was high in eight patients (38%) and CRP in six (28%).

### Medical treatment

The myositis was treated from presentation with oral corticosteroids with the exception of 3 patients. These 3 were initially treated with intravenous methylprednisolone (30 mg/kg to a maximum of 1 g) followed by oral corticosteroid; 2 of these children had calcinosis at presentation. Oral corticosteroid was given in a dose of 2 mg/kg (maximum 60 mg) as a daily dose and weaned according to individual response. No patient’s corticosteroids were weaned while the ESR/CRP or muscle enzymes were abnormal. Methotrexate was given to 15 patients (0.6 mg/kg to a maximum of 25 mg/day) and azathioprine to 2 as steroid sparing agents. In a further 2 patients intravenous cyclophosphamide was used, intravenous immunoglobulin in 2 and rituximab in 1 patient. These agents were used to treat severe relapsing muscle disease.

Diltiazem was tried in 8 patients for either the treatment of or prevention of calcinosis. In 2 of these 5 children with pre-existing calcinosis there was improvement with resolution in some areas, but 2 of the 3 to whom it was given prophylactically developed calcinosis. Chloroquine was prescribed in 4 patients for severe skin disease.

### Follow-up and outcomes

At final review, 10 patients (47%) were in remission after an average follow-up time of 3.2 years (range 1–8). This was defined as being clinically asymptomatic and having normal muscle enzymes and ESR and/or CRP. There were 4 patients with chronic disease with multiple relapses and steroid dependence, of whom one subsequently improved when given intravenous immunoglobulin. Two patients had stopped treatment, relapsed, and were being re-treated. A further 3 were lost to follow up and 2 died. The deaths resulted from complications related to the disease (respiratory arrest due to severe muscle weakness in the first, 9 months after first onset of symptoms and interstitial lung disease with pneumonia in the second, 3 months after first onset of symptoms).

## Discussion

African children and adults with JDM have been described in the context of connective diseases; however, we believe this is the first series of JDM in African children. The typical features of JDM were proximal muscle weakness and characteristic rashes, including vasculitic ulcers, presenting either catastrophically or with slowly progressive symptoms [13]. Other features particularly present in our series included other musculoskeletal features such as arthritis, pulmonary disease, and calcinosis. Even if weakness is not a presenting complaint, in 95% of cases it will be present on objective testing. In this series of African children, 24% of children presented with global rather than proximal weakness. These children were younger at presentation with significantly higher ESR and CRP values and were more likely to develop contractures. This global weakness most likely represents severity in these young patients.

Calcinosis (dystrophic calcification) is a frequent and distressing feature of JDM occurring in association with increased local production of TNFα [9,10]. Calcinosis occurs internationally in up to 40% of children [13], but had a cumulative incidence of 71% in our series. Calcinosis is strongly associated with prolonged inflammation, as well as the vascular form of the disease [14], an observation which holds true for this series of African children in which skin vasculitis was significantly associated with calcinosis, especially in the presence of *Staphylococcus aureus*. This subgroup of children, called JDM with vasculopathy has been previously been described to have a poorer prognosis overall in a study in 1983 by Bowyer et al. [14]. This study also identified the time of onset of disease to initiation of therapy as of primary importance in the development of calcinosis, as well as the use of low dose as opposed to high dose corticosteroids. This recommendation to use high dose aggressive corticosteroid therapy and immunosuppressive agents is commonly cited as central to control ongoing inflammation thought to be of prime importance in the development of dystrophic calcification in JDM [13]. However, the use of high dose corticosteroid in this series of African children did not prevent the development of calcinosis, nor were any differences apparent in the time of onset of symptoms to initiation of therapy in those with and without calcinosis. Interestingly, in a recent series from India, despite the authors noting a delay in presentation, only 18.2% developed calcinosis [15]. In Scandinavia, only 20% developed calcinosis in association with long term disease duration [16]. Our series is in contrast to these reports as well as a study from Brazil in which calcinosis was noted only in the more severe cases, and cardiac involvement was identified being independently associated with calcinosis [17]. However, we had only one child with cardiac disease in this series.

Severity of disease in JDM is often associated with high levels of muscle enzymes whereas a lack of calcinosis has been associated with lower CPK levels [18], even though chronic disease is well described as the norm in JDM [19]. The opposite has been true in this series, where the calcinosis group as a whole has far lower levels of CK, even in those who presented early. The persistence of aldolase or LDH, although also lower than the children in whom no calcinosis occurred, would seem to be a better marker that there is ongoing inflammation in these children.

Staphylococcal infections are common in children with JDM, and have also been noted to play a role in dystrophic calcification. Moore et al. noted an association between calcinosis and staphylococcal infections as each child in their study that had calcinosis also developed staphylociocal infections which they attributed to intermittent defective granulocyte chemotaxis. They also noted that these children had prolonged disease courses and raised enzyme levels for prolonged periods of time [20]. In our study, only 7 of the 15 children in the calcinosis group had staphylococcal infections, but they did not have raised enzyme levels for prolonged periods.

The treatment of dystrophic calcification is generally unsatisfactory, with prevention being the aim. While oral corticosteroids as used in this series are the first line for therapy of JDM, in the face of such high levels of calcinosis, IVMP may play a role, as well as the introduction of methotrexate within six weeks of diagnosis [21,22]. Aggressive therapy is also being used internationally now to induce early remission [23]. We have previously introduced methotrexate from six weeks to three months but this is now being used at diagnosis. Other therapies that have been tried for calcinosis include diltiazem, aluminum hydroxide, probenicid and Bisphosphonates [24].

## Conclusion

We have described a series of African children with JDM. Whilst our series is small, and our conclusion may be limited by numbers as well as all the problems inherent in a cross sectional study, there have been several differences which are at odds with the literature. Whilst the heterogeneity of this disease extends across populations, differences in the disease course, response to therapy and outcome may be due to genetic and local environmental factors, and this may account for the observed differences, particularly high incidence of calcinosis in our study group.

This group has high levels of vasculitic disease associated with high levels of dystrophic calcification, in which the there are lower levels of muscle enzymes, particular CK. This is strongly suggestive of low grade chronic inflammation in this group, and aggressive immunosuppressant use with high dose corticosteroid, early introduction of methotrexate and the use of cyclophosphamide and intravenous immunoglobulin is now employed. This regimen is also used for the younger children, whom we have shown in this series to be at higher risk of contractures and thus long term disability. Muscle enzymes are also an unreliable marker of disease, and clinical measurement of muscle strength is now used rather than laboratory evidence to measure response to therapy. Our patients, particularly in the face of high levels of calcinosis, are at high risk of the developing Staphylococcus Aureus infection, an observation which has lead our unit to institute the practice of routine cloxacillin prophylaxis. There would thus appear to be a role overall for more rapid and aggressive therapy even in the face of what appears to be mild disease in African children.

## Competing interests

The authors declare that they have no competing interests.

## Authors’ contributions

GF conceived the study, collected data, researched the topic and co-wrote the main body of the article. MT did the statistical analysis and co-wrote the main body of the article. BM collected data and critically reviewed the manuscript and research content. All authors read and approved the final manuscript.

## Authors’ information

GF is currently in private practice as a Paediatric rheumatologist at the Wits Donald Gordon Medical Centre in Johannesburg.

BM is a Paediatric rheumatologist, in both private and state practice in Johannesburg.

MT is Chair of the Division of Rheumatology Internal Medicine, Faculty of Health Sciences, Chris Hani Baragwanath Hospital and the University of the Witwatersrand, Johannesburg, South Africa. He has an active interest in paediatric rheumatology.
